# Assessing and Monitoring Nutrition Security in the United States: A Narrative Review of Current Measures and Instruments

**DOI:** 10.1007/s13668-024-00547-7

**Published:** 2024-06-25

**Authors:** Emma Kenney, Victoria O. Adebiyi, Hilary K. Seligman, Mariah D. Ehmke, Joanne F. Guthrie, Alisha Coleman-Jensen, Edward A. Frongillo

**Affiliations:** 1 Department of Health Promotion, Education, and Behavior, Arnold School of Public Health, University of South Carolina, Discovery I Building, 915 Greene Street, Columbia, SC 29208 USA; 2 Division of General Internal Medicine and Center for Vulnerable Populations, University of California, San Francisco, CA USA; 3 United States Department of Agriculture, Economic Research Service, Washington, DC USA

**Keywords:** Nutrition security, Assessment, Monitoring, Health, Food security, Brief instruments

## Abstract

**Purpose of Review:**

Because nutrition plays a crucial role in the development of chronic diseases, ensuring nutrition security is important for promoting population health. Nutrition security is defined as having consistent and equitable access to healthy, safe, affordable foods essential to optimal health and well-being. Distinguished from food security, nutrition security consists of two constructs: healthy diets and nutritional status. The study aimed to identify population measures that reflect the important constructs of nutrition security (i.e., healthy diets and nutritional status) to inform U.S. nutrition security assessment and monitoring.

**Recent Findings:**

Through a narrative review conducted across multiple databases, associations between subconstructs of healthy diets and nutritional status were identified. Of the six subconstructs that constitute healthy diets, nutrient adequacy and moderation were most often used to assess and monitor healthfulness of U.S. population diets and were associated with health outcomes. There is little evidence of an association between health outcomes and macronutrient balance or diversity in the U.S. Thirteen instruments were identified as potentially suitable for measuring at least one subconstruct of healthy diet in the population.

**Summary:**

This review highlights the importance of nutrition security in addressing population health challenges. It emphasizes the potential use of multiple instruments and measures to comprehensively monitor population nutrition security and inform intervention strategies. Identifying feasible and practical measures for assessing and monitoring nutrition security is imperative for advancing population health and mitigating the burden of chronic diseases.

**Supplementary Information:**

The online version contains supplementary material available at 10.1007/s13668-024-00547-7.

## Introduction

Nutrition plays an important role in the etiology of several chronic diseases, including obesity, cardiovascular disease (CVD), type 2 diabetes (T2DM), and certain cancers [1]. A healthy diet consists of nutrient-dense foods across all food groups and can help minimize diet-related chronic disease risk [2]. Conversely, less healthy diets consist of high intakes of added sugars, saturated fat, and sodium, and are responsible for almost half of cardiometabolic deaths from heart disease, stroke, and T2DM in the United States (U.S.) [3]. Over 70% of U.S. adults are either overweight or obese [4] and nearly $173 billion a year is spent on health care for obesity [5]. One third of deaths in the U.S. are caused by heart disease or stroke, and the diseases cost our health care system $216 billion per year [6]. More than 37 million Americans have diabetes, and the American Diabetes Association reported the annual economic cost of diabetes in 2022 was $412.9 billion [7].

Recognizing nutrition’s role in the perpetuation of non-communicable diseases has led to the identification of *nutrition security* as key to addressing U.S. population health. Nutrition security is an emerging concept that places improvement of nutrition and health status within a broad population framework. It has been defined as encompassing consistent and equitable access to healthy, safe, affordable foods essential to optimal health and well-being [8]. The White House Conference on Hunger, Nutrition, and Health identified monitoring the role of nutrition to prevent and manage diet-related chronic conditions (i.e. measuring nutrition security) as a priority [9].

Nutrition security assessment and monitoring relies on availability of relevant nutritional data at the population level that highlight the dietary intake and nutritional status of individuals. Appropriate measures and indicators are essential to track the U.S.’s commitment to healthy diets in the population, advocate for healthfulness of diets, and design policies and programs to achieve these objectives. Having regularly available data for monitoring nutrition requires a coordinated system that provides information on diet, nutrition, and related health outcomes using suitable measures and indicators [10].

Nutrition security complements, but is not the same as, food security [11]. Both include a focus on food access across the population to meet energy needs [8], but nutrition security entails that nutritional needs are met alongside food access [8, 11]. Two constructs–healthy diets and nutritional status–form the basis of nutrition security. Six subconstructs support healthy diets: nutrient adequacy, nutrient density, macronutrient balance, diversity, moderation, and safety [11, 12]. The subconstructs of nutritional status are having optimal amounts in body tissues of energy, protein, essential fats, vitamins, and minerals [11]. While this framework defines constructs and subconstructs of nutrition security, we have not yet identified measures and indicators that are recognized to be suitable to assess and monitor nutrition security.

To begin addressing this gap, we must understand the extent to which existing measures and indicators are suitable for population assessment and monitoring of nutrition security generally and among vulnerable sub-populations. To be suitable, measures and indicators for assessing and monitoring nutrition security should meet the following criteria: 1) reflect important subconstructs of nutrition security; 2) associated with health outcomes; and 3) feasible for population assessment and monitoring (i.e., easy to collect and analyze within a short period of time).

The study’s aim was to identify population measures that reflect the fundamental constructs of nutrition security (i.e., healthy diets and nutritional status) to inform U.S. nutrition security assessment and monitoring. Our analysis focused on four research questions:

1. What is the association of subconstructs of healthy diets with subconstructs of nutritional status?
2. What is the association of subconstructs of healthy diets and nutritional status with food security?
3. What is the association of subconstructs of healthy diets and nutritional status with health outcomes?
4. Which simple measures and indicators of nutrition security, and combinations of them, are feasible for population monitoring?

## Methods

### Search Strategy

We conducted a narrative review by searching electronic databases and selecting literature to ensure a comprehensive coverage of relevant information [13]. Between October 2022 and June 2023, we reviewed peer-reviewed original research, systematic reviews, and grey literature in PubMed and Google Scholar to obtain answers to the four research questions. We developed search strategies for research questions 1–3 (Online Resource 1). For research question 4, we searched the National Cancer Institute (NCI) register of validated brief dietary assessment instruments [14].

### Inclusion and Exclusion Criteria

Articles were included for the first three research questions if they met the following criteria: 1) focused on the U.S. population, 2) measured or focused on some aspect of nutrition security as conceptualized by Seligman et al. [11] (Table 1), 3) described or assessed at least one health outcome or food security, and 4) were published between 2012 and 2023. Review articles were included. Articles were excluded if they focused on food safety, a subconstruct of healthy diets, as food safety does not generally pose a widespread threat to population health in the U.S. Articles were also excluded if they focused on the subconstruct of density because it is related to the subconstruct of nutrient adequacy, and therefore it may be assessed instead of or in addition to adequacy [12]. Brief instruments were included if they were developed or validated among adults in the U.S. Brief instruments that focused only on a sub-population of U.S. adults, e.g., racial minorities, were excluded.

**Table 1:** Constructs and subconstructs of nutrition security

**Constructs & subconstruct**	**Definition**
**Healthy diets**	Food constituents consumed
** Nutrient adequacy**	Nutrient intake relative to requirements without excess
** Nutrient density**	Rich in nutrients per energy content
** Macronutrient balance**	Balance of carbohydrates, proteins, and fats
** Diversity**	Diets composed of a variety of foods derived from diverse food groups
** Moderation**	Intake of food and nutrients related to chronic diseases
** Safety**	Free of microbial pathogens, foodborne macro-parasites, toxins, and chemicals
**Nutritional status**	Constituents in the body
** Energy**	Calories
** Protein**	Nitrogenous organic compounds that have large molecules composed of one or more long chains of amino acids
** Essential fats**	Type of fatty acids (e.g., omega-6, omega-3) that the body cannot produce on it’s own, so they must be obtained through the diet
** Vitamins**	Organic compounds essential for normal growth, metabolism, and health
** Minerals**	Inorganic elements essential for body function

### Data Screening

Two co-authors (E.K. & V.O.A.) screened titles and abstracts of articles independently for eligibility. Duplicates were removed. Those that remained eligible went through full-text screening.

### Data Charting

We developed a data-charting table for data extraction for each research question. For research question 1, we reviewed articles reporting the relationship between the subconstructs of healthy diets and subconstructs of nutritional status. Articles were categorized by population, subconstruct of healthy diet, instrument used to measure the subconstruct of healthy diet, subconstruct of nutritional status, and associations between subconstructs of healthy diets and subconstructs of nutritional status.

For research question 2, we reviewed articles examining the relationships among food security, healthy diet, and nutritional status. The information from each article was extracted and categorized by population, subconstruct, food security measure (e.g., U.S. Adult Food Security Survey Module), and associations between subconstructs of nutrition security and food security.

For research question 3, we reviewed articles looking at the relationship between healthy diets, nutritional status, and health outcomes, extracting information separately for subconstructs of healthy diets and subconstructs of nutritional status. For healthy diets, we reviewed relevant articles and extracted information from the following categories: subconstructs measured, health outcomes measured, and associations between the subconstructs of healthy diets and health outcomes. For nutritional status, we extracted information by subconstructs, biomarker (used to measure the subconstruct), health outcome, and associations between the subconstructs of nutritional status and health outcomes.

For research question 4, we reviewed articles on brief dietary assessment instruments to determine how well they measure healthy diets (or diet quality) and their accuracy in assessing dietary data. The criteria for describing and comparing instruments were adopted from the report by the Institut de Recherche pour le Développement on healthy diet metrics [15] and encompasses the following aspects: 1) instrument, 2) assessment of healthy diet subconstruct, 3) length of measure, 4) predictive validity for health outcomes, 5) how well the instrument assesses dietary data, and 6) ease of computation.

## Results

### Associations between Subconstructs of Healthy Diets and Subconstructs of Nutritional Status

Using different dietary assessment instruments, studies among U.S. adults investigated the relationship between some of the subconstructs of healthy diets (diet diversity, moderation, nutrient adequacy), and nutritional status captured via anthropometry (e.g., body mass index, waist circumference, percent body fat); and the subconstructs of healthy diets and specific biomarkers (e.g., serum cholesterol). Healthy diet subconstructs were assessed via different instruments including the Healthy Food Diversity (HFD) index, Healthy Eating Index (HEI) (1995, 2010 and 2015 versions), and Sustainable Diet Index (SDI)-US. Higher scores on the HFD index (which captures dietary diversity) were associated with lower risk of adiposity or obesity [16]. Studies using the HFD index found lower odds of obesity, android-to-gynoid ratio, and fat mass index (FMI), and lower odds of elevated waist circumference among men and women with the highest quality diet compared to the lowest [16, 17]. Higher scores on the HEI (which captures nutrient adequacy and moderation) were associated with reduced risk of abdominal and central obesity, lower percentage body fat, and lower fat mass index [18–20]. A higher SDI score captured nutrient adequacy and was also associated with lower odds of obesity (Table 2). These findings indicate an association between the subconstructs of healthy diets and nutritional status as higher scores on indices of healthy diets are associated with lower risk of adiposity (i.e., obesity and percentage of body fat).

**Table 2:** Associations between subconstructs of healthy diets and subconstructs of nutritional status

**Author(s), Year**	**Population**	**Subconstruct of Healthy Diet**	**Instrument Used**	**Outcome(s) and Subconstruct of Nutritional Status**	**Associations**
Bailey et al. (2015) [18]	Young women ages 17–25	Adequacy Moderation	Healthy Eating Index (HEI)-2010	Percent body fat	Women in the top quartile of HEI-2010 had significantly lower percent body fat than women in the lowest 3 quartiles. These young women (top quartile of HEI-2010) also had 0.37 odds (95% confidence interval, 0.16–0.85) of having body fat > 32%
Jung et al. (2023) [21]	Adults	Adequacy	Sustainable diet index-US (SDI-US)	Obesity - excess energy	Higher SDI-US score was associated with lower odds of obesity
Vadiveloo, Dixon, et al. (2015) [16]	Adult men and women	Dietary diversity	Healthy Food Diversity (HFD) index	Multiple indicators of adiposity including BMI, waist-to-height ratio, android-to-gynoid fat ratio, fat mass index (FMI), and percentage body fat	The US HFD index was inversely associated with most adiposity indicators in both sexes. The odds of obesity, android-to-gynoid ratio > 1, and high FMI were 31–55% lower (P-trend < 0.01) among women in quintile 5 vs. quintile 1 of the US HFD index. Among men, the odds of obesity, waist-to-height ratio $0.5, and android-to-gynoid ratio > 1 were 40–48% lower (P-trend<0.01) in quintile 5 vs. quintile 1 of the US HFD index
Vadiveloo, Parekh, et al. (2015) [17]	Adult men and women	Dietary diversity	HFD index	Waist circumference, serum cholesterol	Adults in the third vs. first US HFD tertile had elevated waist circumference [0.75 (0.66, 0.86] after multivariable adjustment (P-trend < 0.05). The age- and sex-adjusted odds of low serum HDL cholesterol and impaired fasting plasma glucose (P-trend < 0.05) were lower in the highest vs. lowest US HFD tertile but attenuated with multivariable adjustment (P = 0.06 and 0.22, respectively
Xu et al. (2020) [20]	Adult men and women	Adequacy Moderation	HEI-2015	Percent body fat, fat mass index	Diet quality was inversely associated with percentage body fat and fat mass index in both men and women Diet quality was inversely associated with all fat distribution measures in both sexes
Yoshida et al. (2017) [19]	Mexican American adult men & women	Adequacy Moderation	HEI-2010	Central obesity—excess energy	Higher HEI-2010 total score was associated with lower odds of central obesity in Mexican–American men. A higher whole fruit or sodium score was inversely associated with central obesity in men but not in women

### Associations Between Food Security and Nutrition Security

In the U.S., both food-secure and food-insecure individuals have poor diets [22, 23]. For the subconstructs of healthy diets, macronutrient intakes did not differ when comparing food-secure and food-insecure individuals, but food-insecure individuals were at higher risk of inadequacy for some micronutrients. Among food-insecure men and women, lower intake was found for magnesium, potassium, vitamins B6, C, and D, when compared to food-secure men and women [24]. A systematic review of food insecurity and diet quality found no evidence of an association between food insecurity and intake of carbohydrates or protein and limited evidence of adverse associations with total fat or fiber, with mixed evidence concerning intake of saturated fat [25]. There were, however, associations across samples of varying age and sex between food insecurity and inadequate intakes of calcium, magnesium, and zinc (Table 3).

**Table 3:** Associations between food security (FS) and nutrition security

**Author(s), Year**	**Population**	**Subconstruct(s)**	**Measure of FS**	**Association with FS**
Cowan et al. (2020) [24]	U.S. Adults	Adequacy Vitamins Minerals	US Household Food Security Survey Module	Among food-insecure men and women, the prevalence of inadequate intake was found for magnesium, potassium, vitamins A, B6, B12, C, D, E, and K. No differences in the prevalence of risk for inadequacy were observed for calcium, iron (examined in men only), choline, or folate by food security status
Dhurandhar (2016) [26]	U.S. population	Energy	N/A (review article)	There is evidence that low food security is associated with a weight gain response in the presence of high calorie, energy dense foods in humans. Longitudinally, being marginally food insecure or food insecure without hunger is associated with greater weight gain compared to being fully food secure. Low food security is particularly associated with obesity when Food Assistance programs are in place, and the relationship between Food Assistance programs and obesity is greatest in those who are most food insecure
Eicher-Miller and Zhao (2018) [27]	U.S. children and adolescents	Adequacy, Moderation	N/A (systematic review—multiple methods)	Evidence for a strong, consistent and dose–response relationship of food insecurity with lower vegetable intake compared with food security was determined among children aged 1–5 years and strong and consistent evidence of higher added sugar intake among food-insecure children aged 6–11 years compared with food-secure children was apparent. Adolescent-focused evidence was sparse but revealed adolescence as the pediatric age stage where food insecurity has the most potential for negative impact on child dietary intake
Franklin (2012) [28]	U.S. population	Energy	N/A (review article)	Overall, the review confirmed that food insecurity and obesity continue to be strongly and positively associated in women
Gowda et al. (2012) [29]	U.S. Adults	Blood samples (CRP, white blood cell count, folate, vitamin B12, vitamin A, & cotinine)	US Household Food Security Survey Module	Food insecurity was associated with higher levels of C-reactive protein (adjusted odds ratio [AOR] = 1.21; 95% confidence interval [CI] = 1.04, 1.40) and of white blood cell count (AOR = 1.36; 95% CI = 1.11, 1.67). White blood cell count partly mediated the association between food insecurity and C-reactive protein
Hanson and Connor (2014) [25]	US adults (> = 18)	Overall diet quality (high HEI score – adequacy, moderation, density)	N/A (systematic review—multiple methods)	Four studies examined measures of overall dietary quality on 6 samples or strata. Three nationally representative samples showed an adverse association between food insecurity and overall dietary quality in adults aged 18–64 y, women aged 19–55 y, and one sample of adults aged ≥ 60 y. Two other samples of adults aged ≥ 60 y showed no association, nor did one locally representative sample of adults aged ≥ 18 y
		Macronutrients	N/A (systematic review—multiple methods)	There was no evidence of any association between food insecurity and intake of carbohydrates or protein and very limited evidence of adverse associations with total fat or fiber. There was limited and mixed evidence concerning intake of saturated fat. One nationally representative sample of adults reported an adverse association, whereas 2 other nationally representative samples showed beneficial associations in low-income women and older adults (60–96 y of age)
Jun et al. (2021) [30]	US children (1–18)	Vitamins	US Household Food Security Survey Module	There was no evidence of associations between food insecurity and intake of thiamin or vitamin B-12. There was only very limited evidence to suggest adverse associations between food insecurity and intake of folate, niacin, riboflavin, vitamin C, or vitamin E. However, there was more evidence in women of adverse associations between food insecurity and intakes of vitamin A and vitamin B-6
Lee et al. (2021) [31]	Korean and U.S. adults	Minerals	Household Food Security/Hunger Survey Module (NHANES)	There was no evidence of associations between food insecurity and intake of iron or sodium and only very limited evidence to suggest an adverse association between food insecurity and intake of phosphorus. There was substantial evidence across samples of varying age and sex of adverse associations between food insecurity and intakes of calcium, magnesium, and zinc
Shaheen et al. (2021) [32]	US adults with T2DM	Adequacy, Moderation, Density (HEI)	Adult household food security score (NHANES)	About 24% consumed a poor quality diet and had food insecurity. Those with both factors had higher odds of both an HbA1c 8- < 9% (AOR = 6.1, 95% CI = 1.5–24.8, p = 0.01) and HbA1c ≥ 9% (AOR = 6.7, 95% CI = 2.0–22.2, p < 0.01)
Sharpe et al. (2016) [33]	SC households	Diet quality (AHEI) Adequacy, Moderation, Density	Six-item short form of the 12-month Food Security Scale	Significant dietary differences were few (FS > FI on protein and lean meat; FS < FI on carbohydrate intake). For 29 of 35 (74%) dietary intake recommendations, less than 75% of women in both groups met each recommendation

The literature provides limited evidence regarding associations between the subconstructs of nutritional status and food security. In some studies, U.S. adults show positive associations between food insecurity and obesity, with stronger associations observed in women [26, 28, 34]. Researchers investigating the connection between food insecurity and inflammation found that food insecurity was linked to higher levels of C-reactive protein with an adjusted odds ratio of 1.21 [29]. Furthermore, a study focusing on the association of food insecurity with nutritional biomarkers in U.S. children revealed no difference in vitamin D, zinc, and iron status between food-secure and food-insecure children [30]. The associations between the subconstructs of nutritional status and food security have not been extensively studied in U.S. adults.

### Associations Between Subconstructs of Healthy Diets and Nutritional Status with Health Outcomes

#### Healthy Diets

The adequacy and moderation subconstructs of healthy diet are associated with health outcomes (Table 4). There is less evidence of an association between macronutrient balance or diversity and health outcomes. Most U.S. studies relied on the HEI and Alternate Healthy Eating Index (AHEI) to measure the healthfulness of diets. These indices, along with several others, are primarily constructed to assess adherence to a particular diet (e.g., Dietary Approaches to Stop Hypertension (DASH) score) or dietary guidelines (e.g., HEI). They also encompass measures of adequacy, moderation, and density. The AHEI was developed to better predict health outcomes when compared to the HEI [35–37], but there were no important differences between the associations from the HEI and AHEI with health outcomes [38, 39]. Both the HEI and AHEI are correlated with chronic diseases. Higher scores on both indices are associated with reduced risk of all-cause mortality, cancer, CVD, T2DM, and other chronic diseases (Table 4). The Dietary Patterns Methods Project synthesized that higher diet quality as measured via the HEI, AHEI, alternate Mediterranean diet score, and the DASH score was associated with an 11–28% reduced risk of death due to all-cause, CVD, and cancer when compared to the lower diet quality [40].

**Table 4:** Associations between subconstructs of healthy diets and health outcomes

**Author(s), Year**	**Subconstruct(s)**	**Health Outcomes**	**Associations**
Al-Ibrahim and Jackson (2019) [38]	Adequacy, Moderation, Density	Diabetes (nondiabetes, prediabetes, T2DM)	Neither total HEI-2010 nor AHEI-2010 scores were significantly associated with diabetes status; neither index was clearly superior to the other in terms of its predictive ability in relation to T2DM
Aljuraiban et al. (2020) [39]	Adequacy, Moderation, Density	Cardiovascular disease (CVD) risk	Reductions in CVD mortality associated with the highest and lowest HEI and AHEI scores were 15% and 26%, respectively, although the risk of CVD mortality in obese women was not reported, raising issues regarding generalizability
Asghari et al. (2017) [41]	Adequacy, Moderation, Density Diversity	Obesity	HEI was found to be inversely associated with obesity and diversity-based indices were positively associated with obesity
Bromage et al. (2021) [42]	Adequacy, Diversity, Moderation	Diet related non-communicable disease (NCD) risk	GDQS performed comparably with the Minimum Dietary Diversity—Women indicator in anthropometric and biochemical indicators of undernutrition (including underweight, anemia, and serum folate deficiency). It also performed comparably or better than the AHEI—2010 in capturing NCD-related outcomes (including metabolic syndrome, change in weight and waist circumference, and incident T2DM)
Dietary Guidelines Advisory Committee (2020) [43]	Macronutrient Balance	CVD risk	Limited evidence suggests non-energy restricted diets based solely on macronutrient distribution with either carbohydrate, fat, and/or protein proportions outside of the Acceptable Macronutrient Distribution Range, are neither beneficial nor detrimental regarding risk of CVD in adults, primarily among those at high-risk, such as those with overweight, obesity or features of metabolic syndrome
Gil et al. (2015) [44]	Adequacy, Moderation, Density	Body mass index (BMI) “self-perception” of diet Inflammation Endothelial dysfunction	HEI was correlated positively and significantly with most nutrients in the diet, with BMI of study subjects, & with the individuals “self-perception” of their diets. Higher AHEI scores were associated with lower concentrations of biomarkers of inflammation and endothelial dysfunction and therefore may be a useful tool for reducing the risk of diseases involving such biological pathways
Harrison et al. (2020) [45]	Adequacy, Moderation, Density	Metabolic Syndrome	Higher AHEI and HEI was associated with lower odds of MetS
Liese et al. (2015) [40]	Adequacy, Moderation, Density, Diversity	Mortality from all-causes, CVD, and cancer	Higher diet quality (top quintile) from HEI, AHEI, alternate Mediterranean Diet, & DASH were significantly and consistently associated with an 11–28% reduced risk of death due to all causes, CVD, and cancer compared with the lowest quintile
Miller et al. (2020) [46]	Adequacy, Moderation, Density, Diversity	NCD	Four NCD dietary metrics (MED, HEI, AHEI, & DASH) had convincing evidence of protective associations with specific NCD outcomes, mainly mortality, CVD, T2DM, and cancer
Onvani et al. (2017) [47]	Adequacy, Moderation, Density	Mortality from all-causes, CVD, and cancer	The highest level of adherence to the HEI & AHEI was significantly associated with a reduced risk of all-cause mortality, CVD mortality and cancer mortality
Panizza et al. (2018) [48]	Adequacy, Moderation, Density	Mortality from all-causes, CVD, and cancer	High HEI-2015 scores were inversely associated with risk of mortality from all-cause, CVD, and cancer for men and women
Petersen and Kris-Etherton (2021) [49]	Adequacy, Moderation, Density, Diversity	CVD	Higher diet quality, regardless of the a-priori index used, is associated with a 14–29% lower risk of CVD and 0.5–2.2 years greater CVD-free survival time
Savarino et al. (2021) [50]	Macronutrient balance (child feeding)	Obesity	An unnecessary consumption of high protein formula milks during the first 2 years of life has been associated to later obesity and overweight in pediatric age
Schwingshackl and Hoffmann (2015) [51]	Adequacy, Moderation, Density, Diversity	All-cause mortality, CVD, T2DM, cancer	Diets of the highest quality, as assessed by the HEI, AHEI, and DASH score, resulted in a significant risk reduction for all-cause mortality, CVD, cancer (incidence or mortality), and T2DM
Steck et al. (2015) [52]	Adequacy, Moderation, Density, Diversity	Colorectal cancer	Higher Mediterranean Diet Scores (MDS) were associated with an 8–54% lower colorectal cancer risk. Higher HEI scores were associated with a 20–56% lower colorectal cancer risk. More proinflammatory diet scores were associated with a 12–65% higher colorectal cancer risk compared with more anti-inflammatory diets in studies that used the DII
Verger et al. (2023) [15]	Diversity, Adequacy	NCD	The ability of dietary diversity indicators (DDIs) to reflect diet quality was found to be principally limited to nutrient adequacy, as high diversity reflected nutrient adequacy. DDIs do not readily relate to health outcomes as findings are inconsistent
Wabo et al. (2022) [53]	Macronutrient balance (protein-to-carb ratio (%E P:C))	All-cause mortality	Low %E P:C was associated with an increased risk of death from all-cause mortality

#### Nutritional Status

Energy, a subconstruct of nutritional status, is associated with health outcomes. Excess energy has been linked with adverse health outcomes including obesity, CVD, T2DM, cancer, osteoporosis, and osteoarthritis [54]. Most adults obtain sufficient protein through their diets. There is insufficient evidence to suggest that excess protein intake leads to adverse health effects [55]. There is evidence, however, that long-term consumption of red meat and processed meat is associated with an increased risk of total mortality, CVD, colorectal cancer, and T2DM [56, 57]. Diets high in saturated fats are linked to higher mortality rates from all causes, CVD, and cancer. In contrast, diets rich in polyunsaturated and monounsaturated fats are associated with lower all-cause mortality [58].

The Dietary Guidelines for Americans identified nutrients or vitamins and minerals of public health concern for individuals aged one year and older. The nutrients of concern include low intakes of fiber, calcium (for individuals aged 2 years and older), vitamin D, and potassium. Excessive nutrient intake is a concern for sodium, added sugars, and saturated fats (for individuals aged 2 years and older). These nutrients have been associated with adverse health consequences [59]. For sodium, high intakes above recommendations have been associated with higher risks of hypertension and cardiovascular diseases [60, 61]. Both suboptimal and excessive intakes of nutrients such as calcium, iron, vitamin D, and potassium are associated with health outcomes like hypertension and coronary heart diseases [62–64] (Table 5). For instance, suboptimal vitamin D concentrations are associated with higher risks of CVD, hypertension, cancer, T2DM [63]; while excessive intake of calcium supplements is associated with increased risk of cardiovascular diseases, including coronary heart disease and myocardial infarction [64, 65].

**Table 5:** Associations between subconstructs of nutritional status, biomarkers, and health outcomes

**Author(s), Year**	**Nutrient/Subconstruct**	**Biomarker**	**Associations with Health Outcomes**
Energy			
Marra et al. (2019) [66]	Body Composition	Bioimpedance analysis (BIA)	Useful parameter in clinical practice as it allows identification and monitoring of patients at risk of impaired nutritional status and decreased survival, such as HIV/AIDS, cancer, anorexia, liver cirrhosis, hemodialysis, and pulmonary disease geriatric and surgical patients
		Dual energy X-ray absorptiometry (DXA)	Assessment of bone health to establish diagnosis of osteoporosis and fracture risk requires DXA for evaluating bone mineral density (BMD) in selected anatomical regions of interest
Protein			
Yu et al. (2020) [67]	Protein	Fasting plasma glucose	High-protein diet does not significantly improve glycemic control and blood pressure, but can lower low-density lipoprotein, total cholesterol, triglyceride, and homeostatic model assessment for insulin resistance levels in T2DM patients
Shams-White et al. (2017) [55]		Review article looking at multiple bone biomarkers	Current evidence shows no adverse effects of higher protein intake. There were positive trends on bone mineral density at most bone sites, but only the lumbar spine showed moderate evidence to support benefits of higher protein intake
Hedrick et al. (2012) [57], Richi et al. (2015) [56]	Red meat & processed meat	^13^C	Long-term consumption of increasing amounts of red meat and particularly of processed meat is associated with an increased risk of total mortality, CVD, colorectal cancer and T2DM
		^15^N	
		Creatinine	
		Taurine	
		1-methylhistidine	
		3-methylhistidine	
Fat			
Calder et al. (2017) [68]	Total Fat	Fatty acid desaturase 1 (FADS1) polymorphisms	Associated with a reduced CVD risk
Hedrick et al. (2012) [57]; Kim et al. (2021) [58]		Red blood cell, plasma phospholipids, & cholesterol esters	Diets high in saturated fat were associated with higher mortality from all-causes, CVD, and cancer, whereas diets high in polyunsaturated fat were associated with lower mortality from all-causes, CVD, and cancer. Diets high in trans-fat were associated with higher mortality from all-causes and CVD. Diets high in monounsaturated fat were associated with lower all-cause mortality
Hedrick et al. (2012) [57]; Naghshi et al. (2021) [69]	Essential Fatty Acids (EFA)	Alpha-linolenic acid (ALA) & linoleic acid	Dietary ALA intake is associated with a reduced risk of mortality from all causes, CVD, and CHD, and a slightly higher risk of cancer mortality, whereas higher blood levels of ALA are associated with a reduced risk of all cause and CHD mortality only
Hedrick et al. (2012) [57]	Eicosapentaenoic acid (EPA) and Docosahexaenoic acid (DHA)	eicosapentaenoic acid (EPA)	Intake of omega-3 fatty acids, (EPA) and (DHA) have been linked with reduced CVD risk
		docosahexaenoic acid (DHA)	
		Omega-3 Index	
		N-EPA	
		N-DHA	
Calder et al. (2017) [68]	Inflammation	C-reactive protein (CRP)	Elevated CRP concentrations in “healthy” people are a marker of chronic low-grade inflammation and associated with higher future risk of CVD morbidity and mortality
Vitamins			
Tanumihardjo et al. (2016) [70]; Zabetian-Targhi et al. (2015) [71]; Huang et al. (2021) [72]	Vitamin A	Serum Retinol-Binding Protein (RBP)	RBP4 seems to be correlated with cardiometabolic markers in inflammatory chronic diseases, including obesity, T2DM, metabolic syndrome, and CVDs
		Serum/Plasma Retinol	Higher serum retinol is associated with experienced significantly lower overall, CVD, heart disease, and respiratory disease mortality in a cohort of men
CDC (2012) [73]; Sakakeeny et al. (2012) [74]; Obeid et al. (2019) [75]	Vitamin B6	Serum pyridoxal-5’-phosphate (PLP):	Low vitamin B-6 status, based on plasma concentrations of pyridoxal-5-phosphate (PLP), has been identified in inflammatory diseases, including CVD, rheumatoid arthritis, inflammatory bowel disease, and diabetes
		Serum or urinary 4-Pyridoxic acid (4PA)	High 4-Pyridoxic acid/pyridoxine ratio is independently associated with global CVD risk
Allen et al. (2018) [76]; Wolffenbuttel et al. (2020) [77]; Kweon et al. (2021) [78]; Wang et al. (2022) [79]; Azzini et al. (2021) [80]	Vitamin B12	Serum/plasma B12	In the general population of NHANES, low serum B12 concentrations were associated with a moderate increase in all-cause mortality. There was a small but significant increase in CVD mortality in the groups with low or high serum B12
		Serum holotranscobalamin (holoTC	Measurements of serum holoTC levels combined with total B12 and homocysteine levels may provide valuable information for predicting ischemic stroke and its severity
		Serum methylmalonic acid (MMA)	Elevated serum MMA levels were independently associated with the presence of CVDs and may be used to predict the occurrence of CVDs
		Plasma total homocysteine (tHcy):	There is a direct relationship between elevated fasting plasma levels of homocysteine and several pathological disorders, including bone health, neurodegenerative disease, and renal dysfunction
CDC (2012) [73]; Gibson (2024) [81]; Tian et al. (2022) [82]	Vitamin C	Total ascorbic acid in serum or plasma	Serum vitamin C levels lower than the threshold value (1.06 mg/dL) were negatively associated with all-cause or CVD mortality while in a US population. In contrast, serum vitamin C levels higher than the threshold value were positively associated with all-cause and CVD mortality
Gibson (2024) [81]; Grant et al. (2022) [63]	Vitamin D	Serum 25-hydroxy­vitamin D	Optimal concentration of serum 25-hydroxy­vitamin D above 30 ng/mL (75 nmol/L) has been found to be protective against CVDs
Bailey et al. (2015) [18]; Gibson (2024) [81]; Liu et al. (2022) [83]	Folate	Serum folate concentration	Maintaining moderate levels of serum folate may decrease the risk of CVD death among patients with T2DM
		Red blood cell (RBC) folate concentration	A dose–response relationship has been confirmed between RBC folate concentrations in mothers and risk of neural tube defects in their infants
		Plasma homocysteine concentration	With poor folate intake or impaired folate metabolism, plasma homocysteine level is elevated and has been associated with an increased risk of hypertension, CVD, and cerebrovascular disease
Minerals			
Lynch et al. (2018) [62]; Gibson (2024) [81]	Iron	Serum Ferritin	Low serum ferritin is diagnostic of iron deficiency anemia
		Serum transferrin receptor (sTfR)	Serum trans­ferrin acts as a negative acute phase protein, decreasing in the anemia of chronic disease, whereas in iron defi­ciency anemia, serum trans­ferrin concen­trations are increased
Rohner et al. (2014) [84]; Inoue et al. (2018) [85]	Iodine	Urinary Iodine Concentration	Participants with excess iodine exposure (urinary iodine concentration ≥ 400 μg/L) were at higher risk for all-cause mortality compared to those with adequate iodine nutrition
King et al. (2015) [86]; Qu et al. (2020) [87]	Zinc	Plasma or serum zinc concentration	Elevated serum zinc levels were significantly associated with an increase in total spine and total femur bone mineral density (BMD). Every 10 μg/dL increase was associated with a 1.12-fold increase in diabetes mellitus (DM) and 1.23-fold and 1.29-fold increase in CVD and coronary heart disease (CHD), in participants with serum zinc levels ≥ 100 μg/dL
Michos et al. (2021) [65]; Yuan et al. (2022) [64]; Gibson (2024) [81]	Calcium	Serum calcium	Excessive intake of calcium supplements is associated with increased risk of cardiovascular diseases, including coronary heart disease and myocardial infarction
Jackson et al. (2018) [88]	Potassium	Urinary potassium excretion	An inverse association was found between urinary potassium excretion and blood pressure among US adults
Wang et al. (2020) [60]; Filippini et al. (2022) [61]	Sodium	Urinary sodium excretion	A meta-analysis of cohort studies found an excess risk of hypertension starting at 3 g/day, using dietary intake or urinary sodium excretion of 2 g/day as the reference category Another meta-analysis found that individuals with high sodium intake compared with individuals with low sodium intake had a higher adjusted risk of cardiovascular disease
Wu et al. (2015) [89]; Jawhara et al. (2019) [90]	Fiber	(1) Alkylresorcinol in plasma (2) avenacosides in urine (3) benzoxazinoid-derived phenylacetamide sulfates in blood and urine samples	A significant dose response relationship was observed between fiber intake and the incidence and mortality of coronary heart disease. Consumption of dietary fiber is inversely associated with risk of coronary heart disease, especially for fiber from cereals and fruits

### Summary of Instruments

Twenty-four-hour dietary recall is a standard method to obtain dietary data but is difficult to collect and analyze for timely population-level monitoring. Thus, instruments that measure the relevant subconstructs of healthy diets and nutritional status and are valid and reliable on a population basis are needed for timely population-level assessment and monitoring of nutrition security. The following is a list of potential instruments that may be used to assess and monitor the subconstructs of nutrition security.

#### Healthy Diets

We identified 13 brief dietary instruments out of 63 brief dietary instruments in the National Cancer Institute registry that measured at least one subconstruct of healthy diets and can be used for population assessment and monitoring (Table 6). Three brief dietary instruments developed and validated for global use were also included.

**Table 6:** Brief dietary assessment instruments

**Author(s), Year**	**Measure**	**Subconstruct(s)**	**Information on measure**	**Predictive validity for health outcomes**	**How well does instrument assess dietary data**	**Ease of computation**
NCI (2021) [91]	Dietary Screener Questionnaire in NHANES 2009–10	Adequacy Moderation	- 26 items - asks about the frequency of consumption in the past month of selected foods and drinks - Each of the items on the instrument was selected because of its relationship to one or more dietary factors of interest in dietary guidance	Not assessed	Validity testing has been done for 9 components of the questionnaire—the screener was administered, and the results compared to multiple 24-h recalls. The DSQ and its scoring algorithms produced estimates of mean intake and prevalence that agreed closely with those from multiple 24-h recalls. Differences in the means were < 2%; differences in prevalence were < 16%	The scoring algorithms convert screener responses to estimates of dietary intake for the different food categories
NCI (2021) [91]	Dietary Screener Questionnaire in 2015 National Health Interview Survey (NHIS)	Adequacy Moderation	- 26 items - asks about the frequency of consumption in the past month of selected foods and drinks - captures intakes of fruits and vegetables, dairy/calcium, added sugars, whole grains/fiber, red meat, and processed meat	Not assessed	Not assessed	The scoring algorithms developed for the NHANES DSQ were the same for the NHIS DSQ
NCI (2021) [91]; Thompson et al. (2002) [92]; Greene et al. (2008) [93]	Fruit and Vegetable Screener in Eating at America’s Table Study (EATS)	Adequacy Moderation	- 19 items - 10 food groups - frequency of consumption of specific foods from several food groups	Not assessed	Estimated correlations between the instrument and true intake (from 24-h recall) and between the complete FFQ and true intake were very similar. For the adult population studied, the variation in intakes appears to be captured as well with a short, targeted list of foods without portion size information as with a longer list with portion size information. The instrument might be useful to estimate median intakes of fruit and vegetable servings in US populations but might be less useful in accurately ranking individuals. (Thompson et al. [92])	Different computation for All-Day Screener vs. By-Meal Screener. An algorithm for converting the frequency information is used in conjunction with an algorithm for converting the portion size information to Pyramid/MyPyramid servings
NCI (2021) [91]; Alcantara et al. (2015) [94]	Percentage Energy from Fat Brief Screener	Macronutrient balance	- 14 questions - Short assessment instrument to estimate an individual's usual intake of percentage energy from fat - Asks about the frequency of consumption of 14 specific foods from several food groups, and the average consumption of fat - The foods asked about on the instrument were selected because they were the most important predictors of variability in percentage energy from fat among adults	Not assessed	Mean percentage of energy from fat was 35.5% as reported from the 24 h dietary recall, compared with 33.0% as measured by the NCI fat instrument. The deattenuated Pearson correlation was 0.38, also with notable variation by weight status, education level and age	Algorithm used to convert an individual’s responses to an estimate of that individual’s percentage of energy from fat
NCI (2021) [91]	Multifactor Screener in the Observing Protein and Energy Nutrition (OPEN) Study	Adequacy Macronutrient Balance	- Items from 16 food categories - Intakes of fruits and vegetables, percentage energy from fat, and fiber	Not assessed	Validated against multiple 24-h recalls. Validation results suggest that dietary exposure estimates computed from the Multifactor Screener may be useful to compare subgroup means, especially for populations consuming mainstream diets	Scoring procedures were developed to convert a respondent's screener responses to estimates of individual dietary intake for percentage energy from fat, grams of fiber, and servings of fruits and vegetables
BRFSS Alcantara et al. (2015) [94]; Serdula et al. (1993) [95]	Fruit and vegetable screener	Adequacy	Asks 6 questions on the frequency of intake of fruits and vegetables in the past 30 days	Looking at population-attributable risk, eating < 5 fruits and vegetables based on BRFSS scores was associated with risk of cognitive impairment (7.7%), high cholesterol (11.4%) (Adams et al. [96])	Mean fruit and vegetable intake reported from the 24 h dietary recall was 2.66 servings/d compared with 2.79 servings/d with the BRFSS measure. The deattenuated Pearson correlation was 0.22, with notable variation by weight status, education level and age	Only measures frequency of intake, not portions
England et al. (2015) [97]; Rifas-Shiman et al. (2001) [98]	PrimeScreen	Adequacy	- Instrument (15 items + 8 items on supplement use) - Within each food group daily or weekly	Designed for known relationships between dietary factors and major causes of morbidity and mortality in the USA (i.e., CVD, cancer and osteoporosis)	For foods and food groups, the mean correlation coefficient (r) was 0.70 for reproducibility and 0.61 for comparability with the SFFQ. For nutrients, the mean r was 0.74 for reproducibility and 0.60 for comparability with the SFFQ	Based on the results of the follow-up attitude survey, over 90% of participants found PrimeScreen easy or very easy to understand and complete. The median time to complete PrimeScreen was 5 min; 87% of participants required fewer than 10 min
Fung et al. (2018) [99]; Brennan et al. (2022) [100]	Prime Diet Quality Score (PDQS)	Adequacy Moderation	- 21 food groups and ranges from 0–42 points - Points were assigned according to the following criteria: 0–1 serving/wk (0 point) compared with 2–3 servings/wk (1 point) compared with ≥ 4 servings/wk (2 points) for the healthy food groups	The PDQS was significantly associated with ischemic heart disease in all 3 cohorts separately and the pooled RR for each SD increase was 0.89 (95% CI: 0.87, 0.91)	Spearman correlation coefficients indicated that total PDQS score from PDQS was statistically significantly correlated with total PDQS score from food diaries at months 0 (r = 0.59, p < 0.01) and 3 (r = 0.65, p < 0.01), with similar associations observed via ICCs at months 0 (0.70 (0.49–0.81)) and 3 (0.73 (0.41–0.86))	Scoring was reversed and points deducted for the unhealthy food groups. Points for each food group were then summed to give an overall score
Segal-Isaacson et al. (2004) [101]	The Rapid Eating and Activity Assessment for Patients Survey (REAP-S)	Adequacy Moderation	- 16 items - At least 4 food groups	Not assessed	Compared to the FFQ, The REAP-S appears to be useful for estimating servings of fruits, vegetables, milk, and fat consumption. It also seems to be useful as a rough indicator of fat, cholesterol, fiber and sugar intake	Not assessed
Murphy et al. (2001) [102]	Food Behavior Checklist (FBC)	Adequacy Moderation	Consists of 39 food behavior items, addressing the following topics: 14 items addressing fat/cholesterol intake; 11 items addressing fruit/vegetable intake; 1 item addressing other fiber intake; 2 items addressing dairy products; 6 items addressing overall dietary quality; 2 items addressing food expenditures; and 1 item for salt intake	Not assessed	Criterion validity was tested by comparing with serum carotenoid level. Convergent validity was tested by comparing with multiple 24-h recalls. Responses to 10 food behavior items were significantly correlated with serum carotenoid levels. An additional 12 items showed hypothesized associations with the 24-h recall data. Cronbach’s coefficient alpha ranged from 0.28 (for 5 fat and cholesterol items) to 0.79 (for 9 fruit and vegetable items)	Not assessed
Paxton et al. (2011) [103]	Starting The Conversation (STC) FFQ	Adequacy Moderation	8-item simplified food frequency instrument that identifies dietary patterns designed for use in primary care and health-promotion settings. Items ask about the frequency of consumption of fast food, fruits/vegetables, beans, and fat sources	Not assessed	STC items and the summary score were moderately intercorrelated (r 0.39–0.59, p 0.05). The STC summary score was significantly correlated with the NCI fat screener at baseline (r 0.39, p 0.05), and change in the STC summary score correlated with reduction in percentage of calories from fat (r 0.22, p 0.05) from baseline to 4 months. Overall, the brief eight-item STC tool identified healthful and unhealthful dietary behaviors in a diverse sample, indicating the measure’s feasibility for use in public health and primary care settings	Easy to compute the total score
Block et al. (2000) [104]	Rapid food screener	Adequacy Moderation	One-page food screener based on national nutrition data with two sections. The Meat/Snacks section is comprised of 15 items designed to capture dietary fats. The Fruit/Vegetable section is comprised of 7 items designed to capture fruit and vegetable intake, fiber, and micronutrients	Not assessed	Estimates of fat, fiber, and fruit/vegetable intake derived from the screener were compared with estimates from a full-length, 100-item validated questionnaire. The screener was effective in identifying persons with high-fat intake, or low–fruit/vegetable intake. They found correlations of 0.6–0.7 (p,0.0001) for total fat, saturated fat, cholesterol, and fruit/vegetable intake	The screener can be completed and scored in 5 min or less, and does not require difficult calculations or computer analysis
Bromage et al. (2021) [42]	Global Diet Quality Score (GDQS)	Adequacy Diversity Moderation	- The GDQS is a food-group-based metric of diet quality that was developed for diverse context - The GDQS is composed of 25 food groups considered as important contributors to nutrient intake and/or NCD risk	Not assessed	Performed well compared with the MDD-W in capturing Adequacy, and anthropometric and biochemical indicators of undernutrition	Ease of computation of the GDQS appears to be greatly improved when using the GDQS app
Herforth (2020) [105]; Verger et al. (2023) [15]	Diet Quality Questionnaire Indicator: Global Dietary Recommendations (GDR) Score	Adequacy Density Diversity Moderation	- The DQQ gathers information on consumption (yes/no) of 29 food groups in the previous day - The GDR score is based on the consumption of 17 food groups during the past day and night - The GDR score is a measure of the adherence to a dietary pattern respecting the global dietary recommendations -The GDR score is composed of 2 subscales (the GDR-Healthy score and the GDR-Limit score) and is calculated as follows: GDR-Healthy—GDR-Limit + 9 = GDR score	The GDR score is a measure of the adherence to 11 global dietary recommendations which include dietary factors protective against non-communicable diseases	GDR score was replicated in 10 other countries and appears to be a valid indicator of meeting WHO healthy diet recommendations	The GDR score was developed as a simple, rapid and feasible standardized metric that can be used for surveillance across diverse contexts
Women’s Dietary Diversity Project (2017) [106]	Minimum Dietary Diversity for Women	Diversity Adequacy	- MDD-W is a food group indicator, based on reported intake of 10 food groups	Not assessed	Not assessed	The ease of computation of the MDD-W is facilitated by the fact that only data on whether a food group is consumed (or not) is required

These brief instruments have between six and 26 items administered as a short food frequency questionnaire, food checklist, or behavioral questionnaire about dietary practices. Most instruments measure frequency of consumption of specific foods or food groups in the past month [14]. An exception is PrimeScreen, an instrument that captures the frequency of consumption of certain food groups over the previous year using five categories of frequency of consumption: less than once per week, once per week, two to four times per week, nearly daily or daily, or twice or more per day [97, 98].

While some brief instruments were inclusive of food groups or dietary components [14], others were specific to fruits and vegetables [94]. For example, the Dietary Screener Questionnaire (DSQ) is a 26-item instrument to capture intakes of fruits and vegetables, dairy/calcium, added sugars, whole grains/fiber, red meat, and processed meat. The Behavioral Risk Factor Surveillance System (BRFSS) fruit and vegetable brief instrument, however, has just 6 items on fruit and vegetable intake frequency.

Some brief instruments (e.g., DSQ) have been used in national surveys like the National Health and Nutrition Examination Survey (NHANES) and the National Health Interview Survey (NHIS) [14]. The Prime Diet Quality Score (PDQS), Global Diet Quality Score (GDQS), and Global Dietary Recommendation (GDR) score have been used for global assessment and to distinguish between healthy and unhealthy components using difference scores (i.e., scores that combine both positive (healthy) and negative (unhealthy) components of an instrument to create a total score). Others (e.g., Starting the Conversation or Rapid Eating and Activity Assessment for Participants-Short) were designed and validated for clinical settings [101, 103].

Most brief instruments were validated against standard food frequency questionnaires and/or 24-h recalls to determine how well they measured dietary data (Table 6). Correlations with the standard instruments were reported. While some instruments had modest correlations with standard instruments [94, 97], others underreported some dietary components compared to standard instruments [14]. Few of the identified brief instruments have been examined for predictive validity for health outcomes [96].

The three brief instruments developed for global use measure more healthy diet subconstructs compared to other brief instruments. The GDQS measures nutrient adequacy, diversity, and moderation [42], whereas the GDR score measures nutrient adequacy, nutrient density, diversity, and moderation [105]. The GDQS can be collected using a full 24-h recall but does not require quantifying and converting foods into nutrient values, making data cleaning and processing simpler. The instrument can also be used via an electronic app that provides standard, easy-to-use methods for collecting low-cost, time-relevant data on diet quality. For the ease of data collection, the GDR score relies on a questionnaire that gathers simple information on consumption (yes/no) of a limited number of food groups (or sentinel foods representing those food groups), which allows a short interview time (from 3 to 6 min) and does not require interviewers to have specific training [15].

#### Nutritional Status

There are a range of methods for measuring the subconstructs of nutritional status, including anthropometric, body composition, clinical, and biomarker measures. These include several different techniques for measuring body composition, including anthropometry, dual-energy X-ray absorptiometry (DEXA), and bioelectrical impedance analysis (BIA). DEXA and BIA are valid measures of body composition at the population level, but not feasible for population data collection [54, 66]. Body mass index (BMI) is a widely used anthropometric measure related to body composition that predicts mortality of adults at the population level, but BMI does not differentiate between body lean mass and body fat mass [107–109]. Potential biomarkers for assessment of red meat are 13C, 15N, Creatinine, Taurine, 1-methylhistidine, and 3-methylhistidine [56, 57]. For fats, it may be useful to measure polyunsaturated fats, monounsaturated fats, and saturated fats via red blood cell, plasma phospholipids, and cholesterol esters [57, 58].

Calcium levels in the blood are responsive to hormonal regulation rather than dietary intake. Therefore, monitoring serum calcium levels will not accurately capture changes in dietary calcium intake. Dietary calcium intake is related to bone health. Measuring biomarkers of bone remodeling (e.g., tartrate-resistant acid phosphatase, procollagen type, osteocalcin) should determine the role of dietary intake of calcium on bone health at the population level [81]. It may also be appropriate to assess dietary intake of calcium to assess adequacy of calcium intake. For vitamin D**,** serum 25-hydroxy vitamin D level is an appropriate biomarker to assess vitamin D levels in the blood, as the levels are reflective of dietary intake of vitamin D and sun exposure. This biomarker is not appropriate to assess bone loss due to deficient vitamin D intake [81, 110]. For potassium, blood (serum) potassium concentrations and hypokalaemia are not reliable measures of usual potassium intake or status in the healthy population. Therefore, assessing dietary intake of potassium is more appropriate to determine adequacy of potassium intake [111]. Assessing urinary excretion of sodium, especially over a 24-h period, is an appropriate biomarker for dietary sodium intake at the population level [112]. Lastly, higher consumption of heme iron is associated with higher levels of serum ferritin and hemoglobin levels. Serum ferritin as a biomarker reflects adequate dietary intake of iron, especially heme iron, and is appropriate to assess adequacy of iron intake at the population level [62].

## Discussion

Nutrition plays a pivotal role in the prevention and management of many chronic diseases, prompting the emergence of nutrition security as an essential component in improving population health. Distinguished from food security, nutrition security encapsulates not just access to food but ensuring that food accessed and consumed meets nutritional needs, which is the basis for the subconstructs of healthy diets and nutritional status [11]. To inform U.S. nutrition security measurement needs, our study aimed to identify measures at the population level that reflect important subconstructs of healthy diets and nutritional status. To assess key subconstructs for population monitoring, we reviewed associations between healthy diet and nutritional status, as well as their associations with food security and health outcomes. These findings then laid the foundation for identifying feasible measures and indicators crucial for assessing and monitoring nutrition security.

Some biomarkers might be useful in monitoring nutrition security. For some nutrients, like iron, assessing only subconstructs of healthy diets is sufficient because biomarkers (e.g., serum ferritin) are associated with dietary intake. For other nutrients such as calcium, biomarkers (e.g., serum calcium) are not associated with dietary intake; nevertheless, it may be useful to monitor some biomarkers such as those of bone remodeling that are related to calcium functions in the body, specifically bone health and potentially predictive of health outcomes such as osteoporosis. Measuring all the subconstructs of healthy diets and nutritional status may be ideal but is not practical for population-level monitoring due to time and financial constraints.

Food security is associated with some, but not all subconstructs of nutrition security. For example, in the U.S., limited evidence links food insecurity to specific macronutrient intake differences, although studies highlight associations between food insecurity and lower intakes of calcium, magnesium, and zinc across various age groups and genders. Research in U.S. adults indicates positive association between food insecurity and obesity in some sub-populations and with higher levels of C-reactive protein. While there are associations between food security and certain subconstructs of nutrition security, monitoring both will be important. Nutrition security offers insights that food security alone cannot provide.

Of the six subconstructs identified to constitute healthy diets [11], our review found that nutrient adequacy and moderation were most often used to assess and monitor healthfulness of U.S. population diets and were associated with health outcomes. There is little evidence of an association between health outcomes and macronutrient balance or diversity in the U.S. since most research relies on HEI/AHEI and neither HEI nor AHEI captures these two subconstructs. Further research is needed to relate macronutrient balance and diversity to U.S. population health outcomes.

Various data systems and several instruments are used for dietary assessment. Data systems include the NHANES, the NHIS, and the BRFSS. Indices that are used frequently in the U.S. are the HEI and the AHEI. While HEI is obtained through NHANES data, and frequently utilized in research, data from NHANES has been released to the public every other year, and there can be a substantial lag before that data is publicly available. It may be more opportune to utilize HEI data in combination with a brief instrument or instruments that could be incorporated into other surveys (e.g., NHIS), where data may be released more promptly. Possible existing instruments include the GDQS, GDR score, PDQS, and the BRFSS fruit and vegetable instrument. The GDQS, GDR score, and PDQS also measure healthy and unhealthy food consumption. This may inform policies and programs to promote healthier foods and curb unhealthy food consumption. Further research is required to understand the predictive validity of these brief instruments in relation to health outcomes. Additionally, it is essential to investigate possible differences in the association of unhealthy foods (moderation) and health outcomes vs. healthy foods (adequacy) and health outcomes (i.e., risk reduction).

An ideal nutrition security system for population-level assessment and monitoring should encompass several elements to ensure comprehensive and effective tracking and intervention strategies. The first is regular data collection. There is a need for regular and standardized data collection mechanisms to monitor nutrition security across different demographic groups and geographic regions. This may require multiple data sources, including surveys (e.g., NHANES, NHIS), health facilities, school records, and community health workers to gather comprehensive data. There may be value in linking multiple data sources, or at least making use of multiple different sources, as the 1990 National Nutrition Monitoring and Related Research Act did in bringing together multiple sources of data on nutrition monitoring from federal agencies and state and local governments [10]. Rapid data collection and reporting that leverages the use of technology, can help enable timely reporting and interventions.

The next element is the measure of subconstructs of healthy diets and, if appropriate and possible, nutritional status, as well as monitoring health outcomes of importance, including obesity, CVD, T2DM, and other related health conditions. Nutrition-related chronic diseases often have associated biomarkers that are distinct from traditional measures of nutritional status. For instance, blood pressure, a biomarker for hypertension, does not inherently reflect nutritional status or dietary intake. Similarly, biomarkers like blood glucose levels and HbA1C play roles in diagnosing and monitoring diabetes but do not inherently reflect nutritional status or dietary intake. Measures like these may be helpful in monitoring nutrition security because they tell us about important consequences of nutrition-related chronic conditions.

For measures of healthy diets, adequacy and moderation independently and potentially differentially affect health outcomes. Adequacy is focused on having enough important nutrients in the diet, which may decrease risk of particular health outcomes, whereas moderation is focused on moderating the amounts of unhealthy constituents in the diet, which can increase risk of poor health outcomes. It will be important to track metrics for each of these two subconstructs separately rather than using the difference between the two metrics. Using difference scores in dietary data is not ideal due to overestimation of total scores, ambiguous interpretation (i.e., midrange scores that can have various contributing components resulting in different dietary patterns), low reliability, discarded theoretical information, and discriminant validity issues [39, 113, 114].

Another essential element for a nutrition security system is data dissemination and transparency. Nutrition security data and assessment results should be publicly available to encourage transparency and accountability, and will include effective communication of findings to policymakers, healthcare providers, and the public [115, 116]. The last element is regular evaluation and adaptation of the nutrition security system. Incorporating feedback from stakeholders and communities to improve the system over time, along with continuous evaluation of the system’s effectiveness and adaptability to changing circumstances will be necessary to ensure useful assessment and monitoring. By incorporating these elements into a nutrition security system, policymakers and stakeholders can better understand the nutritional needs of the population and implement targeted interventions and policies to improve nutrition outcomes at the population level.

Several lines of research would be particularly helpful in further developing nutrition security monitoring. First among these is how measures and indicators can capture and reflect disparities in nutrition security across diverse populations. Understanding how various factors such as socio-economic status, geographical region, and cultural background intersect with nutrition security is paramount in developing tailored interventions and policies that can effectively target vulnerable sub-populations. Another priority for research will be the ongoing validating and updating of measures to reflect changes in nutrition security over time. It is essential to consider whether these updates should coincide with guidelines, like the Dietary Guidelines for Americans, to ensure that our measures align with the latest nutritional recommendations. Finally, the question of how these measures should be communicated and utilized remains central. Effective communication and utilization are critical in informing policy decisions, interventions, and resource allocation. In this context, the journey to understanding and improving nutrition security is dynamic and evolving and will require continued attention, research, collaboration, and innovation.

## Conclusion

This analysis outlined the relationship between subconstructs of healthy diets, subconstructs of nutritional status, food security, and health outcomes. The interplay among these highlights the complex understanding of nutrition and its impact on population well-being. The pursuit of feasible and practical measures and indicators for nutrition security monitoring and assessment is a critical challenge. These metrics serve as the foundation for evidence-based policy development and program design, enabling monitoring of progress towards ensuring the attainment of healthful diets for all. The complexity of these associations necessitates an ongoing commitment to research, monitoring, and the implementation of data-driven strategies that can ultimately enhance the health and well-being of our communities.

### Supplementary Information

Below is the link to the electronic supplementary material.

Supplementary file1 (DOCX 16 KB)

## Data Availability

No datasets were generated or analysed during the current study.
