# Supplementary material for: Assessing and Monitoring Nutrition Security in the United States: A Narrative Review of Current Measures and Instruments
Source: Curr Nutr Rep. 2024 Jun 25;13(3):639–67. doi: 10.1007/s13668-024-00547-7 (PMC11327197; doi:10.1007/s13668-024-00547-7)
Supplement: Supplementary file 1 — Supplementary file1 (DOCX 16 KB) [file 13668_2024_547_MOESM1_ESM.docx]

**Online Resource 1**

**PubMed Search Strategy**

1. What is the association between subconstructs of healthy diets and subconstructs of nutritional status?

| Category | Line | Query |
| --- | --- | --- |
| Location | 1 | “United States”[Mesh][Title/Abstract] |
| Subconstruct of healthy diet | 2 | “Adequacy”[Title/Abstract] OR “Moderation” [Title/Abstract] OR “Diversity”[Title/Abstract] OR “Macronutrient Balance”[Title/Abstract] |
| Subconstruct of nutritional status | 3 | “Energy”[Title/Abstract] OR “Protein”[Title/Abstract] OR “Fat”[Title/Abstract] OR “Vitamin”[Title/Abstract] OR “Mineral”[Title/Abstract] |
| Combination(s) |  | (1) AND (2) AND (3) |

1. What is the association of subconstructs of healthy diets and nutritional status with food security?

| Category | Line | Query |
| --- | --- | --- |
| Location | 1 | “United States”[Mesh][Title/Abstract] |
| Subconstruct of healthy diet | 2 | “Adequacy”[Title/Abstract] OR “Moderation” [Title/Abstract] OR “Diversity”[Title/Abstract] OR “Macronutrient Balance”[Title/Abstract] |
| Subconstruct of nutritional status | 3 | “Energy”[Title/Abstract] OR “Protein”[Title/Abstract] OR “Fat”[Title/Abstract] OR “Vitamin”[Title/Abstract] OR “Mineral”[Title/Abstract] |
| Food security | 4 | “Food Security”[Mesh] [Title/Abstract] |
| Combination(s) |  | Step 1: (1) AND (2) AND (4)  Step 2: (1) AND (3) AND (4) |

1. What is the association of subconstructs of healthy diets and nutritional status with health outcomes?

| Category | Line | Query |
| --- | --- | --- |
| Location | 1 | “United States”[Mesh][Title/Abstract] |
| Subconstruct of healthy diet | 2 | “Adequacy”[Title/Abstract] OR “Moderation” [Title/Abstract] OR “Diversity”[Title/Abstract] OR “Macronutrient Balance”[Title/Abstract] |
| Subconstruct of nutritional status | 3 | “Energy”[Title/Abstract] OR “Protein”[Title/Abstract] OR “Fat”[Title/Abstract] OR “Vitamin”[Title/Abstract] OR “Mineral”[Title/Abstract] |
| Health outcome | 4 | “Health Outcome”[Title/Abstract] OR “Diabetes Mellitus”[Mesh][Title/Abstract] OR “Cardiovascular Diseases”[Mesh][Title/Abstract] OR “Chronic Diseases”[Mesh] [Title/Abstract] OR “Noncommunicable Diseases”[Mesh] [Title/Abstract] |
| Combination(s) |  | Step 1: (1) AND (2) AND (4)  Step 2: (1) AND (3) AND (4) |
